# Metabolic, behavioral health, and disordered eating comorbidities associated with obesity in pediatric patients: An Obesity Medical Association (OMA) Clinical Practice Statement 2022

**DOI:** 10.1016/j.obpill.2022.100031

**Published:** 2022-08-06

**Authors:** Suzanne E. Cuda, Roohi Kharofa, Dominique R. Williams, Valerie O'Hara, Rushika Conroy, Sara Karjoo, Jennifer Paisley, Marisa Censani, Nancy T. Browne

**Affiliations:** a Alamo City Healthy Kids and Families, 1919 Oakwell Farms Parkway, Ste 145, San Antonio, TX, 78218, USA; b Center for Better Health & Nutrition, The Heart Institute, Cincinnati Children's Hospital Medical Center, 3333 Burnet Avenue, Cincinnati, OH, 45229, USA; c The Ohio State University College of Medicine Center for Healthy Weight and Nutrition, Nationwide Children's Hospital, 700 Children's Drive LA, Suite 5F, Columbus, OH, 43215, USA; d WOW 4 Wellness Clinic/ PCHC, 6 Telcom Drive, Bangor, ME, 04401, USA; e Division of Pediatric Endocrinology, Baystate Children's Hospital Subspecialty Center, 50 Wason Avenue, Springfield, MA, 01107, USA; f Johns Hopkins All Children's Hospital, Pediatric Gastroenterology, 501 6th Ave S St. Petersburg, FL, 33701, USA; g St Elizabeth Physician's Group Primary Care, 98 Elm Street, Lawrenceburg, IN, 47025-2048, USA; h Division of Pediatric Endocrinology, Department of Pediatrics, New York Presbyterian Hospital, Weill Cornell Medicine, 525 East 68th Street, Box 103, New York, NY, 10021, USA; i 25 Andrews Avenue, Falmouth, ME, 04105, USA

**Keywords:** Behavioral health, Comorbidity, Disordered eating, Metabolic, Obesity, Pediatric

## Abstract

**Background:**

This Obesity Medicine Association (OMA) Clinical Practice Statement (CPS) details metabolic, behavioral health, and disordered eating comorbidities associated with obesity in children. This CPS will be followed by a companion CPS covering further comorbidities, including genetics and social consequences related to overweight and obesity. These CPSs are intended to provide clinicians with an overview of clinical practices applicable to children and adolescents with body mass indices greater than or equal to the 95^th^ percentile for their ages, particularly those with adverse consequences resulting from increased body mass. The information in this CPS is based on scientific evidence, supported by the medical literature, and derived from the clinical experiences of members of the OMA.

**Methods:**

The scientific information and clinical guidance in this CPS is based upon referenced evidence and derived from the clinical perspectives of the authors.

**Results:**

This OMA statement details metabolic, behavioral health, and disordered eating comorbidities associated with obesity in children. It provides clinical information regarding identifying and treating metabolic, behavioral health, and disordered eating comorbidities associated with obesity in children over the 95^th^ percentile of weight/height for age.

**Conclusions:**

This OMA clinical practice statement details metabolic, behavioral health, and disordered eating comorbidities associated with obesity in children and provides an overview of current recommendations. These recommendations lay out a roadmap to the improvement of the health of children and adolescents with obesity, especially those with metabolic, physiological, and psychological complications.

## 1 Introduction

The purpose of this CPS regarding metabolic, behavioral health, and disordered eating comorbidities associated with obesity in children is to provide clinicians with tools to clinically assess and manage children with obesity and its associated complications. The OMA is an organization of providers in the field of obesity medicine dedicated to the comprehensive care of patients with obesity. OMA members are physicians, nurse practitioners, physician assistants, and other healthcare providers who take a comprehensive, evidence-based approach to treating obesity. This approach is comprised of the four pillars of nutrition, physical activity, behavior, and medication. While it is hoped many clinicians may find the recommendations in this CPS helpful, the final decision regarding the optimal care of the patient with overweight or obesity is dependent upon the individual clinical presentation and the judgment of the clinician who is tasked with directing a treatment plan that is in the best interest of the patient.

## 2 Metabolic comorbidities associated with obesity in children

### 2.1 Hypertension

#### 2.1.1 Prevalence

Hypertension is a relatively common complication of obesity in children, and an estimated 3.8–24.8% of adolescents with obesity have hypertension [1,2]. In addition, 50% of youth seen at pediatric weight management clinics have elevated blood pressure [3].

#### 2.1.2 Diagnosis

To diagnose hypertension, three separate measurements should be taken at least one week apart, with at least two measurements at each visit [3,4]. If the patient is less than 13 years old, the following can be used for diagnosis [3]:

• Elevated blood pressure (BP): ≥90th and ≤95th percentile or ≥120/80 mm mercury (mm Hg) to < 95^th^ percentile (whichever is lower)
• Stage 1 hypertension: ≥ 95^th^ percentile to < 95^th^ percentile plus 12 mm Hg or ≥130/80 to 139/89 mm Hg (whichever is lower)
• Stage 2 hypertension: >95^th^ percentile plus 12 mm Hg, or ≥ 140/90 mm Hg (whichever is lower)

If the patient is older than 13 years, the following apply [3]:

• Elevated BP: systolic120–129 mm Hg, diastolic <80 mm Hg
• Stage 1 hypertension: systolic 130–139 mm Hg, diastolic 80–89 mm Hg
• Stage 2 hypertension: ≥ 140/90 mm Hg

Obesity is associated with an increased risk for masked hypertension (clinical BP < 95th percentile but ambulatory BP > 95th percentile) [3]. Circadian variability may also be observed; up to 50% of patients do not experience the expected nocturnal BP dip [2].

#### 2.1.3 Evaluation

During evaluation, ambulatory blood pressure monitoring is recommended due to the prevalence of white coat hypertension and masked hypertension [3]. In addition, screening for glucose intolerance and dyslipidemia are recommended, and sleep history should be checked [5]. Further studies are not necessary unless the patient has a history of chronic kidney disease or other renal disorders. The 2017 Clinical Practice Guideline Screening and Management of High Blood Pressure in Children and Adolescents recommends that echocardiography be performed to assess for cardiac target organ damage (left ventricular mass, geometry, and function) at the time of consideration of pharmacologic treatment of hypertension [[2], [3], [4]]. If there is a possible secondary cause, consider a renal ultrasound, electrolytes, complete blood count, creatinine, renin, and aldosterone [3]. Symptoms may include headache, vision changes, and fatigue.

#### 2.1.4 Treatment

The primary treatment for hypertension in pediatric patients with obesity is weight loss: dietary and lifestyle interventions should come before medication [3]. For elevated blood pressure or stage 1 hypertension without symptoms, implement a lifestyle intervention for six months [3]. Dietary intervention may include a low sodium (<1500 mg (mg)/day) or Dietary Approaches to Stop Hypertension (DASH) diet [3]. Physical activity interventions should aim for 30–60 minutes of moderate to vigorous activity for five days or more a week; restrict only patients with stage 2 hypertension from intense static physical activity until blood pressure is controlled [3]. Implement pharmacotherapy if stage 1 hypertension is not controlled after 12 months or if the patient is symptomatic or has stage 2 hypertension (in combination with lifestyle intervention) [3].

##### 2.1.4.1 Pharmacotherapy

When implementing pharmacotherapy to treat hypertension in pediatric patients, begin with a single agent at the low end of the dosing range [3]. Possible agents may include angiotensin-converting enzyme (ACE) inhibitors, angiotensin II receptor blockers (ARBs), calcium channel blockers, or thiazide diuretics [3]. If the patient has both hypertension and type II diabetes mellitus (T2DM), ACE inhibitors or ARBs are the first line treatment. Increase the dose every 2–4 weeks until BP is normalized [3].

#### 2.1.5 The 2017 clinical practice guideline (CPG) for screening and management of hypertension and children with obesity

##### 2.1.5.1 Challenges with the 2017 clinical practice guideline and management of children with obesity

The new normative data were based on normal weight children, thus the cut off values for the categories of “elevated or ≥90^th^ percentile, stage 1 or ≥95^th^ percentile, and stage 2 or ≥95^th^ percentile plus 12 mm of mercury)mm Hg)” are lower than older clinical practice guidelines [4]. This creates a conundrum: the proportion of children with obesity and hypertension is now higher based on the lowering of the normative values.

##### 2.1.5.2 Associations between hypertension and children with obesity

Associations between children with obesity and hypertension include [2,3,5]:

• A graded increase in the rate of hypertension with increasing waist circumference
• Lack of circadian variability of blood pressure: up to 50% do not experience the expected nocturnal dip
• Development of hypertension as an adult
• Hypertension in children with obesity is frequently accompanied by dyslipidemia and disordered glucose metabolism, all of which contribute to greater increases in cardiovascular (CV) risk.

Severe obstructive sleep apnea (OSA) is commonly associated with hypertension in children with obesity [3]. Additionally, in children with T2DM and obesity, progression from normo-tension or prehypertension to hypertension is strongly associated with central obesity [6].

##### 2.1.5.3 General guidelines

Diet and lifestyle interventions are the cornerstone of treatment, but 7% of children with elevated BP may still progress to hypertension [3]. Children with obesity and stage 2 hypertension should be restricted from high static sports (wrestling, weight lifting, boxing, etc.) [3]. Acute, severe hypertension requires immediate evaluation. Ambulatory blood pressure monitoring can be an objective method to evaluate treatment [[2], [3], [4]]. The top takeaways regarding hypertension are shown in Table 1.

**Table 1:** **Top Takeaways: Hypertension.** Shown are the top takeaways from the OMA regarding hypertension and obesity in children.

1.	Hypertension is a relatively common comorbidity of obesity in children, and an estimated 3.8–24.8% of adolescents with obesity have hypertension [1,2].
2.	Three separate blood pressure measurements may be used for diagnosis, with cutoffs depending on the age of the patient.
3.	During evaluation, ambulatory blood pressure monitoring is recommended as well as screening for glucose intolerance and dyslipidemia, and sleep history should be checked.
4.	The primary treatment for hypertension in pediatric patients with obesity is weight loss.
5.	Pharmacotherapy is recommended for persistent stage 1 hypertension despite 6–12 months of dietary therapy or stage 2 hypertension.
6.	Children with obesity and stage 2 hypertension should be restricted from high static sports.
7.	Acute, severe hypertension in children requires immediate evaluation.

### 2.2 Idiopathic intracranial hypertension (IIH)

#### 2.2.1 Diagnosis

Idiopathic intracranial hypertension (IIH) is also known as pseudotumor cerebri (PTC), benign intracranial hypertension (BIH), and pseudotumor cerebri syndrome. IIH affects more female patients than male patients: primary IIH patients are 72.5% female, and secondary IIH patients are 75.8% female [7,7,8]. However, this sex difference changes based on pubertal status; the pre-pubertal female-to-male ratio is 1:1.04, while the post-pubertal female-to-male ratio is 6:1 [9,10]. In addition, higher body mass indexes (BMIs) are noted in post-pubertal patients with primary IIH [7,8,11,12].

IIH can lead to permanent visual impairment or blindness [7,8]. There is a strong association between childhood obesity and increased risk of pediatric IIH. The incidence of IIH in youth with obesity is lower in prepubertal children than in adolescents [9,10].

#### 2.2.2 Evaluation

Idiopathic intracranial hypertension (IIH) is defined as elevated intracranial pressure without clinical, radiological, or laboratory evidence of a cause. Important clinical findings include headaches, visual loss (including blind spots), poor peripheral (side) vision, double vision, and short temporary episodes of blindness [7,13]. A full neurological examination can help evaluate IIH, usually with imaging and a spinal tap. A full eye examination for papilledema or loss of visual field should also be conducted [14,15].

#### 2.2.3 Treatment

Preserving vision and controlling symptoms are the goals of therapy. In general, a variable response to all therapies may be observed. Ventricular/lumbar peritoneal shunts are an option that may lower intracranial pressure, preserve vision, and relieve other symptoms. There are some case reports of successful treatment due to weight loss after Roux-en-Y gastric bypass [16,17]. Loss of as little as 6% of body weight has been noted to reduce intracranial pressure [18]. Pharmacotherapy treatments may include acetazolamide, headache management (which can include topiramate), serial lumbar punctures, and lumbo-peritoneal shunts if needed [[7], [8], [9]].

#### 2.2.4 Clinical findings

In one study of 606 adults with obesity, there was an incidence of 2.8% with abnormalities on non-mydriatic fundus photographs and a 0.6% incidence of asymptomatic optic disc edema [15]. Symptoms included headaches, visual loss, including blind spots, poor peripheral (side) vision, double vision, and short temporary episodes of blindness [15]. The top takeaways from the OMA regarding IIH are shown in Table 2.

**Table 2:** **Top Takeaways: Intracranial Hypertension (IIH).** Shown are the top takeaways from the OMA regarding intracranial hypertension and obesity in children.

1.	There is a strong association between children and adolescents with obesity and increased risk of pediatric intercranial hypertension (IIH).
2.	IIH can lead to permanent visual impairment or blindness.
3.	Important clinical findings include headaches, visual loss (including blind spots), poor peripheral (side) vision, double vision, and short temporary episodes of blindness.
4.	A full neurological examination can help evaluate IIH, usually with imaging and a spinal tap, and a full eye examination for papilledema or loss of visual field should also be conducted.
5.	Loss of as little as 6% of body weight has been noted to reduce intracranial pressure; pharmacological treatments may also be used if deemed appropriate.

### 2.3 Sleep disorder syndromes, sleep guidelines, and sleep hygiene

#### 2.3.1 Sleep hygiene

##### 2.3.1.1 Definition

Sleep hygiene encompasses a variety of practices and habits that are necessary to have good nighttime sleep quality and full daytime alertness [19].

##### 2.3.1.2 Instructions to follow for optimal sleep

For optimal sleep quality, keep bedtimes and wake times consistent every day of the week. Late weekend nights or sleeping in can throw off a sleep schedule for days. Bedtime should follow a predictable sequence of events, such as brushing teeth and reading a story. Having physical exercise early in the day can help with sleep time. Worry time should not be at bedtime. Children with this problem can try having a “worry time” scheduled earlier to think about and discuss their worries with a parent. Security objects at bedtime are often helpful for children who need a transition to feel safe. When checking on a child at night, checks should be “brief and boring” [19].

##### 2.3.1.3 Things to avoid

Avoid high stimulation activities just before bed, such as watching television or playing video games. Also avoid spending non-sleep time in bed. This includes situations where a child is awake in bed tossing and turning; in this case, have the child get out of bed to do a low stimulation activity (e.g., reading), then return to bed later. Do not consume caffeine (sodas, chocolate, tea, coffee) in the afternoons or evenings. For the best night's sleep, most adults and children should avoid strenuous workouts close to bedtime [19].

#### 2.3.2 Guidelines for sleep duration

Table 3 shows National Sleep Foundation recommendations for duration of sleep based on age.

**Table 3:** **Guidelines for Sleep Duration.** Shown are recommendations for sleep duration (in hours) based on age from the National Sleep Foundation [20].

	National Sleep Foundation Recommendation (hours)	Not Recommended (hours)
Newborn	14–17	<11 or >19
Infants	12–15	<10 or >18
Toddler	11–14	<9 or >16
Preschool	10–13	<8 or >14
School-Aged	9–11	<7 or >12
Teenager	8–10	<7 or >11
Young Adult	7–9	<7 or >9
Adult	7–9	<7 or >9
Older Adult (65+)	7–8	<7 or >8

#### 2.3.3 Sleep disorder syndromes

Several sleep disorder syndromes exist that may impact pediatric patients with obesity. Table 4 shows some of these syndromes as well as their diagnoses, evaluations, treatments, and clinical findings.

**Table 4:** **Sleep Disorder Syndromes.** Shown are common sleep disorder syndromes and their diagnoses, evaluations, treatments, and clinical findings.

	Diagnosis	Evaluation	Treatment	Clinical Findings
Primary Snoring	Prevalence: 3–16% [21,22] Snoring without apnea or hypopnea, hypercapnia, hypoxemia, high arousal index, disruption of normal sleep architecture or daytime symptoms [22]	Sleep study if severe to rule/out apnea or hypopnea, hypercapnia, hypoxemia, high arousal index, disruption of normal sleep	Sleep hygiene counseling	Decrease in mean verbal intelligence quotient (IQ) scores, global IQ scores, selective attention scores, sustained attention scores, memory index [23]. Direct correlation between number of mild desaturations (3%)/arousals & severity of neurocognitive deficits [23] Characterized as benign; linked to neuropsychological impairments.
Restless leg syndrome (RLS)	Insomnia with “creepy or crawling” feeling in the arms and legs at night Insomnia accompanied by an urge to move the limbs [21]	Diagnosis of exclusion No single test can diagnose RLS Low iron levels are suggestive Electromyography (EMG) and possible muscle biopsy to rule out other neuro-muscular disease [21,22]	Sleep hygiene counseling Dopamine agonists Oral iron Clonazepam Gabapentin [21,22]	Autosomal dominant, sensorimotor disorder 2/3 of children may have low levels of serum ferritin; iron is a cofactor in synthesis of dopamine. Restless legs syndrome and attention deficit disorder are associated. Interferes with going to sleep and staying asleep May be accompanied by daytime fatigue, inattentiveness, or frank sleepiness. Dopamine deficiency implicated in the pathogenesis [21,22]
Delayed Sleep Phase Syndrome	Habitually unable to fall asleep before 2–3 am Neurological sleep disorder in which a person's sleep/wake cycle is delayed with respect to the external day/night cycle Normal sleep if sleeps freely but very sleepy if conforming to conventional sleep-wake schedules [21,22]	History of circadian sleep pattern Perform sleep logs and wrist actigraphy Evaluate for secondary effects of chronically poor sleep [21,22]	“Bright light” therapy: provide 2700–10 000 lux of bright light via a light box for 20–30 minutes immediately after waking leading to a gradual phase advancement (shifting back) of the sleep onset time at night. 0.5–1 mg melatonin about 5.5 hours before sleep onset Stimulant drugs to counter residual daytime sleepiness [21,22]	Habitually unable to fall asleep before 2–3 am Prefer to wake in the late morning or early afternoon. Higher likelihood of the presence of the Human Leukocyte Antigen (HLA) DR1 on chromosome 6, and the occasional familial clustering of delayed sleep phase syndrome suggest a genetic predisposition. Delayed sleep phase syndrome linked to polymorphisms in Period (Per) gene. Onset: typically, adolescence, boys > girls [21,22]
Obesity Hypoventilation Syndrome (OHS)	Awake chronic hypercapnia; partial pressure carbon dioxide (PaCO_2_) >45 ​mm Hg) ​+ ​Obesity Excessive work of breathing and increased carbon dioxide (CO_2_) production Abnormal central ventilatory drive and obesity Other disorders should be ruled out Also known as Pickwickian Syndrome [21,22,24,25]	Evaluate for concurrent obstructive sleep apnea (OSA) (90% chance) Sleep study: Oxygen (O_2_) Saturation levels, CO_2_ levels, and apnea episodes Cardiac and pulmonary work up if symptomatic [21,22,24,25]	Weight loss Respiratory support as indicated, including continuous positive airway pressure (CPAP) [21,22,24,25]	May have central respiratory control system abnormality with decreased responsiveness to CO_2_ rebreathing, hypoxia, or both. Inspiratory muscle strength and resting tidal volumes decreased Leptin deficiency/resistance may contribute to OHS by reducing ventilatory responsiveness leading to CO_2_ retention. Pulmonary hypertension (HTN) more common & severe than in OSA. [21,22,24,25]

#### 2.3.4 Obstructive sleep apnea

##### 2.3.4.1 Diagnosis

In patients with obstructive sleep apnea (OSA), the upper airway becomes blocked repeatedly during sleep, reducing or completely stopping airflow [22]. OSA may be a cause of obesity and not a consequence alone [22,26,27]. The STOP-Bang Questionnaire is a clinical OSA screening instrument for adults that assesses risk and aids diagnoses by evaluating relevant factors: **s**noring, **t**iredness, **o**bservations (of choking or stopping breathing while sleeping), high blood **p**ressure, **b**ody mass index, **a**ge (older than 50), **n**eck size, and **g**ender [28]. Additionally, a modified version of the STOP-Bang instrument may be used to assess OSA risk in adolescents [29].

##### 2.3.4.2 Evaluation

The apnea-hypopnea index (AHI) can be used to determine the presence and severity of OSA in sleep studies [30]:

• AHI 1–1.5 = mild
• AHI 1.5–5.0 = moderate
• AHI >5.0 = severe

Evaluation by an ear, nose, and throat specialist (ENT) for obstruction can also determine the presence of OSA. Additionally, titration of O_2_ saturation can be used for evaluation, and one may consider electrocardiogram/echocardiogram and ferritin levels. Assess blood pressure in patients with OSA; this is a feature of metabolic syndrome which, in combination with OSA, increases cardiovascular risk [[31], [32], [33], [34], [35], [36], [37]].

##### 2.3.4.3 Treatment

Treatment for OSA includes tonsillectomy and adenoidectomy (T&A) if indicated. Repeat the sleep study ≥ 6–8 weeks post-op. Weight loss is a recommended treatment for OSA (other recommended treatment should be used pending weight loss), including implementing a routine sleep pattern [38,39].

##### 2.3.4.4 Clinical finding

The history of patients with OSA may include [29,40]:

• Snoring or disrupted sleeping
• Daytime sleepiness
• Hyperactivity
• Audible pauses in breathing
• Nocturia

The top takeaways from the OMA regarding sleep disorder syndromes, sleep guidelines, and sleep hygiene are shown in Table 5.

**Table 5:** **Top Takeaways: Sleep Disorder Syndromes, Sleep Guidelines, and Sleep Hygiene**. Shown are the top takeaways from the OMA regarding sleep disorder syndromes, sleep guidelines, and sleep hygiene in children.

1.	Sleep hygiene encompasses a variety of practices that are necessary to have good nighttime sleep quality and full daytime alertness, including consistent sleep times and wake times, consistent bedtime routines, and avoidance of stimulation before sleep.
2.	Recommended sleep durations vary based on age, with younger children generally needing more sleep.
3.	Several sleep disorder syndromes exist that may impact pediatric patients with obesity, including primary snoring, restless leg syndrome, delayed sleep phase syndrome, and obesity hyperventilation syndrome.
4.	In patients with obstructive sleep apnea (OSA), the upper airway becomes blocked repeatedly during sleep, reducing or completely stopping airflow.
5.	A modified STOP-Bang instrument can be used to assess OSA risk in adolescents, and the apnea-hypopnea index (AHI) can be used to determine the presence and severity of OSA in sleep studies.
6.	Weight loss is a recommended treatment for OSA, and tonsillectomy and adenoidectomy may be used if indicated.

### 2.4 Prediabetes and diabetes

#### 2.4.1 Prediabetes

##### 2.4.1.1 Diagnosis

Fasting blood glucose (FBG) is a common diagnostic tool to assess for diabetes and prediabetes; FBG ≥100 mg/dl but <126 mg/dl on repeat measurements indicates prediabetes (impaired fasting glucose). In addition, a hemoglobin A1c (HbA1c) ≥ 5.7% but <6.5% after two measurements or 2-h oral glucose tolerance test (OGTT) for blood glucose ≥140 mg/deciliter (mg/dl) but <200 mg/dl (impaired glucose tolerance) are also utilized in the diagnosis of prediabetes [[41], [42], [43]].

##### 2.4.1.2 Evaluation

Similar to diagnosis, evaluation of disease includes FBG and 2-h OGTT. In addition, consider the fasting insulin of the patient; it may be falsely low if the disease is severe or mildly elevated during pubertal growth spurts. Major clinical predictors of prediabetes to type 2 diabetes mellitus (T2DM) progression include severe obesity, impaired glucose tolerance, and ethnicity. High risk ethnic groups include African Americans, Mexican Americans, Native Hawaiians, American Indians, Pacific Islanders, and Asian Americans [41,42,44].

##### 2.4.1.3 Treatment

A restricted carbohydrate diet is often helpful in treating prediabetes. Adolescent studies have shown increases in insulin sensitivity with intensive diet- and exercise-induced weight loss. Consider treatment with metformin for patients with HbA1c ≥ 5.8% when compliant with diet [45,46].

##### 2.4.1.4 Clinical findings

Prediabetes is a reversible, intermediate phase of altered glucose metabolism in the progression from normal glucose tolerance to T2DM. Acanthosis nigricans, or hyperpigmentation in axillae, umbilicus, groin, and popliteal fossae, often occurs in patients with prediabetes and diabetes [41,44,47].

#### 2.4.2 Type II diabetes mellitus

##### 2.4.2.1 Diagnosis

Diagnosis for type II diabetes may include HbA1c ≥ 6.5%, FBG ≥126 mg/dl (fasting is defined as no caloric intake for at least 8 hours), 2-h oral glucose tolerance test (OGTT) for blood glucose ≥200 mg/dl (the test should be performed as described by the World Health Organization (WHO), using a glucose load containing the equivalent of 1.75 milligram/kilogram (mg/kg) [max 75 g] anhydrous glucose dissolved in water), or classic symptoms of hyperglycemia and a random glucose measurement of >200 mg/dl [43,[48], [49], [50]].

##### 2.4.2.2 Evaluation

In children with diabetes mellitus, HbA1c should be measured every three months. Individualize home self-monitoring and provide comprehensive diabetes self-management education and support. Assess the patient's social context and address any mental/behavioral health/disordered eating that is present. Consider the patient's weight when recommending medications. Provide preconception counseling for females starting at puberty. Measure pancreatic autoantibodies, including glutamic acid decarboxylase 65-kDa isoform (GAD65) and islet antigen 2 (IA2; insulin autoantibody if no exposure to exogenous insulin) as well as the urine albumin/creatinine ratio (UACR) and alanine transaminase (ALT) and aspartate aminotransferase (AST) at baseline [43,[48], [49], [50]]. These tests confirm the diagnosis of Type 2 vs Type 1 diabetes and help to define the status of renal and hepatic complications.

##### 2.4.2.3 Treatment

Treatment for children and adolescents with obesity and T2DM includes comprehensive lifestyle management aiming to achieve a 7–10% decrease in body weight. If HbA1c is <8.5%, metformin is the initial pharmacotherapy choice. If FBG is ≥ 250mg/dl or HbA1c is ≥ 8.5%, treat with basal insulin while metformin is being initiated and titrated [51]. Current recommendations state that the initial treatment of youth-onset T2DM should include metformin and/or insulin alone or in combination; the decision should be based on the metabolic status of patient [51]. In 2019, the use of the glucagon-like peptide 1 (GLP-1) receptor agonist liraglutide was approved to treat T2DM in children ≥10 years. If the patient is ketotic or ketoacidosis is present, use subcutaneous or IV insulin to correct the hyperglycemia. If the patient is presenting with severe hyperglycemia (FBG ≥600 mg/dl), assess for hyperosmolar hyperglycemic nonketotic coma (HHNK) [43,[48], [49], [50]].

##### 2.4.2.4 Management

Treat BP greater than the 95^th^ percentile with an ACE inhibitor if there is no response to lifestyle management after 6 months. If there is a worsening urine albumin-to-creatinine ratio.

(UACR), refer the patient to nephrology. Screen for neuropathy annually with foot examination. Additionally, screen for retinopathy both at diagnosis and annually, and screen for symptoms of OSA at each visit [43,[48], [49], [50]].

##### 2.4.2.5 Type II diabetes mellitus in youth: specific issues

There was a dramatic increase in youth onset of T2DM in the late 1990s [52]. Prevalence has been shown to increase with age, tripling from 10–14 years to 15–18 years of age. Progression to an HbA1c ≥ 8% occurs in 46.6% of patients if treated with metformin plus lifestyle intervention. Pancreatic beta cell function declines at 20–35% per year. HbA1c ≥ 6.3% after 3 months of metformin predicts loss of glycemic control [52].

Several large studies in youth-onset type 2 diabetes have occurred in recent years, including the SEARCH for Diabetes in Youth study, the TODAY (Treatment Options for type 2 Diabetes) study and RISE (Restoring Insulin Secretion) study [[53], [54], [55], [56]].

There are two overall forms of T2DM in youth: one that is easily controlled and one that is rapidly progressive. Non-modifiable risk factors include a strong family history of T2DM in first- or second-degree relatives, offspring of pregnancy complicated by gestational diabetes mellitus (GDM), minority race/ethnicity, and the physiologic insulin resistance of puberty [51]. As compared to adults, insulin sensitivity in adolescents is 50% lower, even when adjusted for body mass index (BMI) [52]. For youth who develop T2DM there is at least a 50% chance that the disease will progress despite treatment [57].

Adolescents hyper secrete insulin at levels 2–3 times the levels in adults. Treatments which are effective in adults, including insulin and/or metformin, are not successful in maintaining beta cell function at 15 months post baseline [58]. Comorbidities include [58]:

• Hypertension: present in 1/3–2/3rds of patients within 1–4 years of onset. Treat upon diagnosis with an ACE inhibitor.
• Dyslipidemia: present in 60–75% of patients; the target low density lipoprotein (LDL) is < 100 mg/dl.
• Retinopathy: present in 9% of patients by their first visit. Conduct an initial dilated comprehensive eye exam and continue every 2–3 years.
• Microalbuminuria: begin screening at diagnosis and repeat annually; treat with an ACE inhibitor if persistent.
• Depression: prevalence is higher in T2DM patients than in those with Type 1 diabetes mellites (T1DM); it is associated with poor adherence to diet and medication. If present, the patient should be referred for mental health care.
• Non-alcoholic fatty liver disease (NAFLD): measure baseline ALT and refer the patient to a gastroenterologist if indicated.
• OSA: perform a polysomnogram if suspected, echocardiogram if present, and treatment if indicated.
• Polycystic ovary syndrome (PCOS): evaluate in female adolescents, including laboratory studies when indicated.
• Dyslipidemia: Lipid testing is to be performed once initial glycemic control is achieved. Lipid testing should be performed annually. The goal is LDL ≤100 mg/dl. If LDL is ≥ 130 mg/dl after 6 months of dietary intervention, a statin should be initiated.
• Skin tags [41,44,47].

The top takeaways from the OMA regarding prediabetes and diabetes are shown in Table 6.

**Table 6:** **Top Takeaways: Prediabetes and Diabetes.** Shown are the top takeaways from the OMA regarding prediabetes, diabetes, and obesity in children.

1.	Hemoglobin A1c (HbA1c) tests are a common diagnostic tool for diabetes and prediabetes; fasting blood glucose (FBG) and 2-h oral glucose tolerance tests (OGTTs) are also common and effective.
2.	Major clinical predictors of progression from prediabetes to type 2 diabetes mellitus (T2DM) include severe obesity, impaired glucose tolerance, and ethnicity; high-risk ethnic groups include African Americans, Mexican Americans, Native Hawaiians, American Indians, Pacific Islanders, and Asian Americans.
3.	A restricted carbohydrate diet is often helpful in treating prediabetes.
4.	Treatment for T2DM includes comprehensive lifestyle management aiming to achieve a 7–10% decrease in excess weight; pharmacological treatments may also be implemented, such as metformin and insulin.
5.	There was a dramatic increase in youth onset of T2DM in the late 1990s, and prevalence has been shown to increase with age.
6.	Non-modifiable risk factors for T2DM include a strong family history of T2DM in first- or second-degree relatives, offspring of pregnancy complicated by gestational diabetes mellitus (GDM), minority race/ethnicity, and the physiologic insulin resistance of puberty.

### 2.5 Dyslipidemia

#### 2.5.1 Diagnosis

The predominant pattern of dyslipidemia in children and adolescents with obesity is elevated triglycerides (TG), low high-density lipoprotein cholesterol (HDL-C), moderately elevated non-HDL-C, and normal to mildly elevated low-density lipoprotein cholesterol (LDL-C) [59]. If the pattern of dyslipidemia is different from this, pursue a workup and treatment per National Heart, Lung, and Blood Institute (NHLBI) guidelines and consider referral to a lipidologist [60]. Elevated or abnormal levels are [59,61]:

• TG > 100 mg/dl if < 10 years, > 130 mg/dl if ≥ 10 years
• HDL-cholesterol <40 mg/dl; total cholesterol >200 mg/dl
• Non-HDL cholesterol ≥145 mg/dl; LDL cholesterol >130 mg/dl

#### 2.5.2 Evaluation

Evaluate the patient's fasting lipid profile and repeat the evaluation every 3–6 months if it is abnormal. You may also monitor the patient using the non-fasting lipid profile if this is more feasible. Examine the patient's family history of premature cardiovascular disease, which may help differentiate dyslipidemia of obesity from heterozygous familial hypercholesterolemia. Consider genetic testing for persistent elevations of LDL >160 mg/dl and/or TG > 500 mg/dl [59,61].

#### 2.5.3 Treatment

Lifestyle modification is the first step in treatment of dyslipidemia. To address triglycerides, decrease sugar intake and consumption of sugary beverages, and increase complex carbohydrate and fiber intake. LDL-C and non-HDL-C may be addressed by reducing saturated fat intake to <10% of total daily intake and increasing fiber intake [59,[62], [63], [64]]. Polyunsaturated or monounsaturated fats may be substituted [65].

#### 2.5.4 Pharmacology

While the first line of treatment is lifestyle modification, if the goal levels are not being achieved, one can consider adjunctive medication. Pharmacology for LDL-C and non-HDL-C includes statins, cholesterol absorption inhibitors, niacin, and bile acid binding resins. Soluble fiber 12 g per day is also useful for treating LDL-C and non-HDL-C. Triglycerides may be addressed by omega-3 fatty acids, niacin, and fibrates. Doses of omega-3 of 1–4 g may be used in children with TG ≥ 500 mg/dl with the purpose of reducing the risk of pancreatitis [59,66,67]. The top takeaways from the OMA regarding dyslipidemia are shown in Table 7.

**Table 7:** **Top Takeaways: Dyslipidemia.** Shown are the top takeaways from the OMA regarding dyslipidemia and obesity in children.

1.	The predominant pattern of dyslipidemia in children and adolescents with obesity is elevated triglycerides (TG), low high-density lipoprotein cholesterol (HDL-C), moderately elevated non-HDL-C, and normal to mildly elevated low-density lipoprotein cholesterol (LDL-C).
2.	Evaluate the patient's fasting lipid profile and repeat the evaluation every 3–6 months if it is abnormal; you may also monitor the patient using the non-fasting lipid profile if this is more feasible.
3.	Lifestyle modification is the first step in treatment of dyslipidemia, including lowering sugar intake and increasing physical activity.
4.	Pharmacological treatment may be useful if goals are not achieved with lifestyle modification; treatments may include statins, cholesterol absorption inhibitors, and bile acid binding resins.

### 2.6 Early cardiovascular disease

#### 2.6.1 Diagnosis

Elevated BMI is a risk factor for early cardiovascular disease (CVD); thickening of the carotid intima is associated with increased BMI [68,69]. If metabolic syndrome (MetS) is used as a predictor of early CVD, individual components are more reliable than presence or absence of MetS. Male sex is a risk predictor for CVD at all weight categories. MetS identifies children 8–14 years of age at increased risk for T2DM, and early subclinical atherosclerosis corresponds to a 15-fold increase in risk [68,70]. Increased left atrial (LA) and left ventricular (LV) mass, as well as epicardial fat, are associated with early CVD. Prepubertal children with obesity have a vascular adaptation state with higher heart rates, greater resting and reactive arterial blood flow, and larger brachial artery diameter than normal weight children. By late adolescence, they develop increased arterial stiffness, suggesting that adaptation fails due to the more severe or longer duration of obesity and/or the onset of puberty [[71], [72], [73], [74]].

#### 2.6.2 Evaluation

Cardiovascular damage is already apparent in childhood based on autopsies. Elevated systolic and/or diastolic blood pressure (>140/90 mm Hg) is associated with early CVD. Increased severity of obesity is correlated with increased CVD risk as measured by components of the metabolic syndrome up until BMI of 140% of the 95th percentile, when risk plateaus. Carotid intima-media thickness (cIMT) of 0.07 mm or greater in children with obesity vs. age/height matched children with normal weight is an indicator of CVD. Maximal or submaximal exercise testing is a strong predictor of CVD. Increased left ventricular mass measured on echocardiography is an independent risk factor of cardiovascular morbidity and mortality [[71], [72], [73], [74]].

#### 2.6.3 Treatment

Treatment for early CVD involves intensive lifestyle modifications. Exercise programs (including aerobic and resistance training) should be implemented. In one study, regular exercise over 6 months restored endothelial function and improved cIMT in children with obesity [75]. Improved cardiorespiratory fitness (CRF) is associated with a reduction in CVD mortality. Although treatment with statins and hypertensive medications in adults is associated with decreased incidence of myocardial infarction (MI) and stroke and predicts declining acceleration of cIMT, no intervention studies with medication have been done in children. Bariatric surgery in adolescents reverses the metabolic complications and cardiac structural and functional changes but there are, as of yet, no reports of the long-term effects on cardiovascular health [76,77]. The top takeaways from the OMA regarding early cardiovascular disease are shown in Table 8.

**Table 8:** **Top Takeaways: Early Cardiovascular Disease.** Shown are the top takeaways from the OMA regarding early cardiovascular disease and obesity in children.

1.	Elevated BMI is a risk factor for early cardiovascular disease (CVD), and thickening of the carotid intima is associated with increased BMI.
2.	Elevated systolic and/or diastolic blood pressure (>140/90 mm Hg) is associated with early CVD, and increased severity of obesity is correlated with increased CV risk.
3.	Treatment for early CVD involves intensive lifestyle modifications with a focus on exercise and cardiorespiratory fitness.
4.	No medication studies have been conducted in children, although some medications have been proven effective in adults.
5.	Bariatric surgery improves metabolic complications and CV structural and functional changes in adolescents, but there are currently no reports on long-term effects on CV health.

### 2.7 Metabolic syndrome

#### 2.7.1 Diagnostic criteria: modified national cholesterol education program adult treatment panel III (NCEP-ATP III)

For ages 12–19 years, diagnostic criteria can include the 2003 Cook criteria with a modification of the fasting blood glucose (FBS) criterion to the 2003 American Diabetes Association (ADA) criterion of ≥100 mg/dl as abnormal. According to these criteria, metabolic syndrome is indicated by the presence of any three of the following [[78], [79], [80]]:

• TG ≥ 100 mg/dl
• HDL-C ≤ 10^th^ percentile for age and sex
• FBS ≥100 mg/dl
• Waist circumference (WC) ≥ 90th percentile for ethnicity, age, and sex
• BP (mmHg) ≥ 90th percentile for age, height, and sex

#### 2.7.2 Diagnostic criteria: International Diabetes Federation

According to the International Diabetes Federation, metabolic syndrome can be diagnosed by central obesity plus two risk factors. For ages 10–16 years, these include [79,80]:

• WC ≥ 90th percentile for age and sex; adult cut-off if lower
• SBP ≥130 or DBP ≥85 mmHg
• TG ≥ 150 mg/dl
• HDL-C< 40 mg/dl
• FBS ≥100 mg/dl

For age >16 years, risk factors include [79,80]:

• WC ≥ 94 cm for males; ≥ 80 cm for females (Asians and Central and South Americans: ≥ 90 cm in males)
• SBP ≥130 or DBP ≥85 mmHg or previously diagnosed hypertension
• TG ≥ 150 mg/dl or treatment for hypertriglyceridemia
• HDL-C < 40 mg/dl for males; <50 mg/dl for females or treatment for low HDL-C
• FBS ≥100 mg/dl or known T2DM

#### 2.7.3 Evaluation

The following can be used to evaluate metabolic syndrome in pediatric patients [[79], [80], [81], [82]]:

• Fasting plasma glucose ≥100mg/dL
• Fasting lipid panel: triglycerides and HDL-C
• Waist circumference (WC)
• Systolic and diastolic blood pressure (SBP and DBP)
• Birth history: metabolic syndrome (MetS) has been associated with increased birth weight, maternal obesity, and gestational diabetes mellitus.
• Children who were large for gestational age (LGA) at birth or who were exposed to an intrauterine environment of diabetes or maternal obesity are at increased risk of metabolic syndrome.

The presence of MetS is a significant predictor of CVD and future type 2 diabetes in children [83,84].

#### 2.7.4 Treatment

Children with lifestyle intervention (physical activity, nutrition education, and behavior therapy) are found to have a significant decrease in MetS prevalence and improvement of blood pressure, waist circumference, and 2-h glucose values on OGTT compared to children with obesity without intervention. The degree of weight loss (body mass index standard deviation score [BMI SDS] reduction >0.5) is associated with an improvement in the prevalence of all MetS components [85,86]. The main approach for dietary changes as recommended by the American Academy of Pediatrics (AAP), the American Heart Association, and WHO includes: 1.) increase in vegetable and fruit consumption 2.) a reduced intake of saturated fat and substitution of unsaturated fats (i.e., olive oil and other vegetable oils) and 3.) reduction in sugar intake [87]. The top takeaways from the OMA regarding metabolic syndrome are shown in Table 9.

**Table 9:** **Top Takeaways: Metabolic Syndrome.** Shown are the top takeaways from the OMA regarding metabolic syndrome and obesity in children.

1.	For ages 12–19 years, diagnostic criteria for metabolic syndrome include the 2003 Cook criteria with a modification of the fasting blood glucose (FBS) criterion to the 2003 American Diabetes Association (ADA) criterion of ≥100 mg/dl as abnormal.
2.	According to the International Diabetes Federation, metabolic syndrome can be diagnosed by central obesity plus two risk factors (listed in section 2.7.2).
3.	The presence of metabolic syndrome is a significant predictor of CVD and future type 2 diabetes in children.
4.	Children with lifestyle intervention (physical activity, nutrition education, and behavior therapy) are found to have a significant decrease in metabolic syndrome prevalence and improvement of blood pressure, waist circumference, and 2-h glucose values on OGTT compared to children with obesity without intervention.

### 2.8 Polycystic ovary syndrome (PCOS)/Menstrual irregularity

#### 2.8.1 Diagnosis of PCOS

Polycystic ovary syndrome (PCOS) may be diagnosed by the presence of oligomenorrhea/amenorrhea and clinical or biochemical hyperandrogenism, with the frequent presence of obesity, glucose intolerance, dyslipidemia, and OSA [88,89]. Pathophysiology involves a combination of factors such as adrenal and ovarian hyperandrogenism, insulin resistance, adiposity, and gonadotropin secretion abnormalities [90]. PCOS can present in both lean adolescents and those with obesity. Not every adolescent with obesity and menstrual irregularity has PCOS. Hirsutism, which may or may not be clinically evident, is also associated with PCOS.

#### 2.8.2 Diagnosis of menstrual irregularity

Excessive weight gain can be associated with menstrual irregularity. Irregular menses is defined as a less-than-21-day or greater-than-45-day interval. Implement treatment for patients with intervals greater than 3-months, and for those with less than 9 cycles in 12 months at a gynecological age greater than 18 months and who are human chorionic gonadotropin (HCG) negative [89,91].

#### 2.8.3 Evaluation

For evaluation, measure prolactin, estradiol, consider luteinizing hormone/follicle stimulating hormone, thyroxine/thyroid stimulating hormone, free testosterone, total testosterone, dehydroepiandrosterone sulfate (DHEA-S), sex hormone binding globulin, and early morning 17-OH progesterone. Implement a medroxyprogesterone challenge if oligomenorrhea is present. Consider the use of a pelvic ultrasound or 2-h OGTT [88,89,91].

#### 2.8.4 Treatment

Treatment is individualized and depends on symptoms. Interventions include metformin, combined oral contraceptive pills, spironolactone, and specific treatments for hirsutism and acne, such as hair removal and topical and systemic medication for acne [90]. Oral contraceptive pills (OCPs) are the first line treatment for most patients. Progestin monotherapy is an alternative if OCPs are contraindicated. Lifestyle modification and dietary control are also helpful treatment methods. Beneficial effects of exercise in adolescents have been found for a range of metabolic, anthropometric, and cardiorespiratory fitness-related outcomes [92]. Weight loss is shown to improve insulin sensitivity and reduce cardiovascular risk. Consider the use of metformin; it is most effective in combination with weight loss. Metformin is considered for abnormal glucose tolerance [91]. The top takeaways from the OMA regarding PCOS and menstrual irregularity are shown in Table 10.

**Table 10:** **Top Takeaways: Polycystic Ovary Syndrome (PCOS)/Menstrual Irregularity.** Shown are the top takeaways from the OMA regarding PCOS and menstrual irregularity in children with obesity.

1.	Polycystic ovary syndrome (PCOS) may be diagnosed by the presence of oligomenorrhea/amenorrhea and clinical or biochemical hyperandrogenism, with the frequent presence of obesity, glucose intolerance, dyslipidemia, and OSA.
2.	Excessive weight gain can be associated with menstrual irregularity, which is defined as a less-than-21-day or greater-than-45-day interval.
3.	For evaluation, measure thyroid stimulating hormone, free thyroxine, prolactin, estradiol, consider luteinizing hormone/follicle stimulating hormone, free testosterone, total testosterone, dehydroepiandrosterone sulfate (DHEA-S), sex hormone binding globulin, and early morning 17-OH progesterone.
4.	Treatment is individualized and will depend on symptoms, with many options available.
5.	Oral contraceptive pills are the first line treatment for most patients, but other interventions include metformin, progestin monotherapy, spironolactone, and specific treatments for hirsutism and acne, such as hair removal and topical and systemic medication for acne.
6.	Lifestyle modification and dietary control are helpful treatment methods.

### 2.9 Orthopedic conditions

Many orthopedic conditions can be associated with obesity in pediatric patients. Table 11 shows some of these conditions as well as their diagnosis, evaluation, and treatment.

**Table 11:** **Orthopedic Conditions.** Shown are the diagnosis, evaluation, and treatment of common orthopedic conditions.

	Diagnosis	Evaluation	Treatment
Blount's Disease [[93], [94], [95]]	• Early walking (before the age of 12 months) in a child with severe obesity • Dome-shaped metaphysis, open growth plate and disruption of the continuity between the lateral borders of epiphysis and metaphysis, with inferomedial translation of the proximal tibial epiphysis	• Anterior/posterior (AP) and Lateral views of the tibia	• Surgical correction
Slipped Capital Femoral Epiphysis (SCFE) [[96], [97], [98]]	• Hip pain or limp: consider referred pain to groin, knee, limb • Male:Female ratio = 1.5:1 • Age of onset Males = 12.7–13.5 years • Age of onset Females = 11.2–12 years • Severe obesity at 5–6 years old: 5.9 times greater risk of SCFE compared with those with a normal BMI • Severe obesity at 11–12 years had 17.0 times the risk of SCFE	• AP and Lateral views of the hips • Ultrasound • Degree of severity depends on avascular necrosis and/or instability	• Surgical emergency (In situ pinning) • Intertrochanteric osteotomy
Scoliosis [99]	• Physical findings may be obscured by obesity • Increased curve magnitude at presentation	• Traditional Adam Forward Bend Test • Shoulder height asymmetry or use of a scoliometer	• Brace vs determine whether curve is great enough to require surgery (>45°)
Pes Planus [100,101]	• More prevalent in children with overweight/obesity compared to normal weight controls; prevalence estimates 4–28%.	• Should be evaluated with the patient standing/weight-bearing • Evaluate for presence of symptoms (i.e., pain, decreased endurance)	• If symptomatic, orthoses to control excessive pronation, stretching exercises

### 2.10 Nonalcoholic Fatty Liver Disease (NAFLD)

#### 2.10.1 Diagnosis

Screen all patients with severe obesity or a family history of NAFLD. Alanine transaminase.

(ALT) levels two times above normal levels indicate NAFLD [102]. ALT greater than 80 is more commonly associated with nonalcoholic steatohepatitis (NASH) [103]. A diagnosis of exclusion can also be used and should rule out genetic/metabolic, medication-related, dietary, and infection-related causes. ALT is currently the best screening test for NAFLD. However, a limitation is that liver function tests (LFTs) can be normal with NAFLD. Persistently elevated ALT warrants further evaluation and an exploration of other causes of chronic hepatitis. Approximately 15% of children with NAFLD have advanced fibrosis at diagnosis. Children with NAFLD should be evaluated for hypertension, dyslipidemia, and T2DM [[104], [105], [106]].

#### 2.10.2 Evaluation

Imaging technology is a top evaluation method for NAFLD. Recommended technologies include [104,[107], [108], [109], [110]]:

• Liver ultrasound: however, this method has suboptimal sensitivity and specificity
• Magnetic resonance imagery (MRI)-proton density fat fraction, which also detects steatosis
• Transient elastography is highly reliable with a low failure rate and high intra- and inter-operator reproducibility for assessment of hepatic fibrosis and steatosis
• Magnetic resonance elastography, which also detects fibrosis

Liver biopsy is considered the gold standard of diagnosis, but it is invasive and costly, making it a non-ideal diagnostic tool. Follow children with NAFLD yearly to monitor the progression of the disease. If the initial evaluation is normal, repeat every 2–3 years for situations in which risk factors are same over time, but repeat more frequently if clinical risk factors increase [111]. The clinician should also screen for other conditions that cause elevated liver enzymes [112].

#### 2.10.3 Treatment

NAFLD treatment requires intensive lifestyle modifications [113]. Nutritional modifications include the removal of sugar-sweetened beverages and other improvements in diet, including reductions in processed and high-sugar foods and reduction of saturated fats [114]. Activity modifications should include increasing moderate-to-high intensity exercise, aiming for an average of 40 minutes for 5 days per week with decreases in screen time [115]. There are currently no available medications or supplements that are recommended to treat NAFLD in pediatric patients. Liraglutide is currently used in adults, but studies in pediatrics are needed [116,117].

If NAFLD is present, optimize control of diabetes mellitus, hypertension, and dyslipidemia. Liver transplant may be required in advanced cases. Metabolic and bariatric surgery may be beneficial, but further study is needed. Metabolic and bariatric surgery may be particularly beneficial in adolescents with BMI ≥35 kilogram/meter^2^ (kg/m^2^) who have noncirrhotic NAFLD and serious co-morbidities [116,118].

#### 2.10.4 Clinical findings

NAFLD is often asymptomatic, but clinical signs may include acanthosis nigricans and hepatomegaly. One in six adolescents with NAFLD have depression, which is correlated with worse liver chemistries; one in eight adolescents with NAFLD have anxiety [104,119].

NAFLD is the most common chronic liver disease in US children. NAFLD occurs in 40% or more of children with obesity. It occurs more frequently in males than in females. There is a higher prevalence in patients who are Hispanic, followed by White, Asian, and Black [120,121].

At-risk populations include those with overweight and obesity, OSA, insulin resistance, prediabetes, diabetes, dyslipidemia, central adiposity, a family history of NAFLD and/or NASH, early onset obesity (before age two), deterioration of nutritional status during first 6 years of life, Hispanic ethnicity, binge eating disorder, and nocturnal eating; eating out is correlated with higher odds of NASH [[121], [122], [123], [124], [125]]. The top takeaways from the OMA regarding NAFLD are shown in Table 12.

**Table 12:** **Top Takeaways: Nonalcoholic Fatty Liver Disease.** Shown are the top takeaways from the OMA regarding NAFLD and obesity in children.

1.	In diagnosis of NAFLD, screen all patients with severe obesity or a family history of NAFLD; alanine transaminase (ALT) levels two times above normal levels suggest NAFLD.
2.	Liver biopsy is considered the gold standard of diagnosis, but it is invasive and costly, making it a non-ideal diagnostic tool; imaging is a top evaluation method for NAFLD.
3.	NAFLD treatment requires intensive lifestyle modifications; there are currently no available medications or supplements that are recommended to treat NAFLD in pediatric patients.
4.	If NAFLD is present, screen for diabetes mellitus, hypertension, and dyslipidemia. Liver transplant may be required in advanced cases.
5.	NAFLD is often asymptomatic, but clinical signs may include acanthosis nigricans and hepatomegaly.
6.	NAFLD is the most common chronic liver disease in US children and occurs in 40% or more of children with obesity.

### 2.11 Vitamin D deficiency

#### 2.11.1 Diagnosis

Deficiency of vitamin D is defined by the Institute of Medicine and Endocrine Society clinical practice guidelines as serum 25-hydroxyvitamin D [25(OH)D] < 20 ng/milliliter (ng/mL).

#### 2.11.2 Significance

Vitamin D is a fat-soluble vitamin essential for skeletal health in growing children. It has an important role in bone health through the absorption of calcium from the small intestine [126]. It is available in some foods and through synthesis from sunlight. Low serum vitamin D status in US adolescents has been found to be strongly associated with hyperglycemia, hypertension, and metabolic syndrome, with a relationship independent of adiposity. Vitamin D deficiency has been found to be involved in inflammatory processes that occur in children with obesity and affects insulin secretion and resistance [127]. A recent meta-analysis of patients with T2DM provided level 1 evidence that vitamin D supplementation may reduce chronic low-grade inflammation. Lower levels of C-reactive protein (CRP), tumor necrosis factor alpha (TNF-alpha), and erythrocyte sedimentation rate (ESR), and increased leptin concentrations, are found in vitamin D supplemented groups [128].

#### 2.11.3 Treatment

In children aged 1–18 years, treatment should include [128,128,129]:

1. 2000 international units (IU)/day of vitamin D2 or vitamin D3 for at least 6 weeks or
2. 50,000 IU of vitamin D2 or D3 once a week for at least 6 weeks to achieve a blood level of 25(OH)D above 20 ng/ml, followed by maintenance therapy of 600–1000 IU/day.

#### 2.11.4 Special considerations

Children with obesity, malabsorptive syndromes, or taking medications affecting vitamin D metabolism (i.e., anticonvulsants, glucocorticoids, antifungals, and antiretrovirals) may require two to three times the dose of vitamin D to achieve the same serum 25(OH)D levels as children without these conditions [130]. The top takeaways from the OMA regarding vitamin D deficiency are shown in Table 13.

**Table 13:** **Top Takeaways: Metabolic Comorbidities Associated with Obesity in Children.** Shown are the top takeaways from the OMA regarding metabolic comorbidities associated with obesity in children.

1.	Low serum vitamin D status in US adolescents has been found to be strongly associated with hyperglycemia, hypertension, and metabolic syndrome, with a relationship independent of adiposity.
2.	Deficiency of vitamin D is defined by the Institute of Medicine and Endocrine Society clinical practice guidelines as serum 25-hydroxyvitamin D [25(OH)D] < 20 ng/mL.
3.	Treatment includes supplementation with vitamin D for at least 6 weeks or until acceptable blood levels are reached.
4.	Children with obesity, malabsorptive syndromes, or taking medications affecting vitamin D metabolism may require larger doses to achieve the same serum levels as children without these conditions.

## 3 Behavioral health comorbidities

### 3.1 Attention-deficit hyperactivity disorder and obesity

The association between attention deficit hyperactivity disorder (ADHD) and obesity has been studied for over 2 decades [131]. ADHD impulsivity and inattention, reward deficiency syndrome, and insufficiency of dopamine suggest an explanation for this association [132,133]. The obesity/ADHD association often co-occurs with the diagnoses of sleep disorders, loss of control eating disorder/binge eating disorder (LOC-ED/BED), and anxiety. This co-occurrence may be explained by shared neuroendocrine pathways [134,135]. Manifestations of ADHD associated with obesity include difficulty in planning as a consequence of ADHD inattentiveness (skipping meals); ADHD impulsivity (eating disorders such as binge eating disorder [BED]); and short sleep duration [136]. ADHD is also associated with reduced levels of physical activity in childhood and associated with obesity in adolescence. ADHD is also highly associated with other psychiatric disorders including seasonal depression, anxiety, commonly seen in children with obesity.

Physical activity can be a mediating factor for both diseases with social play mitigating ADHD symptoms in many children. There is association of un-medicated ADHD with higher BMIs during childhood compared to ADHD treated with stimulants. Starting stimulants at younger age and longer duration is associated with slower BMI growth earlier in childhood but a more rapid rebound to higher BMIs later [137]. When co-occurring with loss of control eating or binge eating disorder, lisdexamfetamine offers dual benefit [138].

### 3.2 Depression

Depression frequently co-exists with obesity in children [[139], [140], [141]]. More severe depression is associated with higher BMI weight categories and is more likely in females than males [142]. The onset of depression and/or obesity is bidirectional. The combination of obesity and depression increases the risk of cardiovascular disease 5-fold [143].

Influences that can raise the risk of obesity and depression include [144]:

• Use of social media associated with an increase in body dissatisfaction
• Peer victimization, bullying and teasing related to weight
• Co-existing eating disorders (ex. binge eating disorder)

The diagnosis of depression is made by a mental health professional through structured interviews and validated depression scales. Treatment is coordinated by a mental health professional, preferably in coordination with the weight management team. Physical activity is beneficial in improving symptoms of depression in children with obesity [145]. Management of depression includes consideration of pharmacotherapy, often antidepressants. Antidepressant usage is independently associated with increasing BMI trajectory over time. The mental health safety of the child takes precedence, but it is also important to consider the effects of any medication on weight. Initial improvement in depression can occur post weight loss from metabolic and bariatric surgery, but symptoms may return with any weight regain. Whatever obesity treatment is employed, mental health support is key in addressing preexisting mental health issues along with support for weight loss/regain adjustment concerns [139,141].

### 3.3 Anxiety

Obesity in children is strongly associated with depression and anxiety even after other risk factors (socioeconomic status, parental depression, other neuropsychiatric disorders) are taken into account [146]. Prevalence in the general population is 20–25% of youth with the median age of onset 6 years [144]. The frequency (odds ratio) of anxiety in children with obesity is 1:30. Children with severe obesity have five times higher risk of anxiety. Anxiety with increased BMI is more common in females than in males [147,148].

Because of increased prevalence, routine screening for anxiety is recommended in children and adolescents with overweight/obesity. Potential screening instruments include [149]:

• Screen for Child Anxiety Related Disorders (SCARED) (41 items-widely used)
• Generalized Anxiety Disorder (GAD-7) (7 items)
• Spence Children's Anxiety Scale (SCAS) (35–45 items)

A child with a positive screen should be referred to a mental health professional. After evaluation, possible treatments include psychotherapy (Cognitive Behavioral Therapy [CBT]; mindfulness-based psychotherapies and psychodynamic psychotherapies [150]. If pharmacotherapy is added to the treatment regimen, selective serotonin reuptake inhibitors (SSRI)s are first line therapeutic choices. Of note, SSRI medications are frequently associated with medication induced weight gain; the choice of SSRI to treat a mental health condition needs to balance the therapeutic effectiveness of the medication with a possible exacerbation of weight gain [151].

### 3.4 Emotional eating

The diagnosis of emotional eating (EE) is characterized by eating in response to negative emotions or stress. EE shifts negative emotions to food cues and is associated with adult and childhood trauma exposure or emotional abuse. Other characteristics of EE are a history of high dietary restraint; decreased awareness of hunger/satiety cues; and emotion dysregulation of the hypothalamic pituitary adrenal (HPA) stress response (the normal response to stress is loss of appetite) [152]. EE may be a mediator between depression and obesity as a coping mechanism to regulate and reduce negative emotions [153].

The prevalence of EE is higher in females than males and has a low prevalence in younger children with the disorder usually emerging in adolescence. EE is associated with depression and anxiety with a genetic predisposition combined with parental psychological control [154].

Tools to evaluate for EE include [144,155,156]:

• Dutch Eating Behavior Questionnaire (DEBQ)
• Child Eating Behavior Questionnaire (CEBQ)
• Emotional Eating Scale for Children and Adolescents (EES-C)

Treatment of EE focuses on emotion regulation skills rather than a calorie-restricted diet. Dialectical behavior therapy (DBT) with modules on mindfulness, emotion regulation and distress tolerance are utilized along with mindfulness-based parent stress intervention [157]. Structured interdisciplinary obesity treatment with nutrition, physical activity, and family counseling/psychosocial support are recommended [150,158]. Table 14 shows the top takeaways regarding behavior health comorbidities associated with obesity in children.

**Table 14:** **Top Takeaways: Behavioral Health Comorbidities Associated with Obesity in Children.** Shown are the top takeaways from the OMA regarding behavioral health comorbidities associated with obesity in children.

1.	The obesity/ADHD association often co-occurs with the diagnoses of sleep disorders, loss of control eating disorder/binge eating disorder (LOC-ED/BED), and anxiety.
2.	Antidepressant usage is independently associated with increasing BMI trajectory over time.
3.	Whichever obesity treatment is employed, mental health support is key in addressing preexisting mental health issues along with support for weight loss/regain adjustment concerns.
4.	Obesity in children is strongly associated with depression and anxiety even after other risk factors (socioeconomic status, parental depression, other neuropsychiatric disorders) are taken into account
5.	SSRI medications are associated with medication induced weight gain; the choice of SSRI to treat a mental health condition needs to balance the therapeutic effectiveness of the medication with a possible exacerbation of weight gain.
6.	EE is associated with depression and anxiety with a genetic predisposition combined with parental psychological control.

## 4 Disordered eating comorbidities

### 4.1 Binge eating disorder

Binge eating disorder (BED) is defined as excessive overeating that feels out of control, becomes a regular occurrence, and is without recurrent inappropriate compensatory behaviors (i.e., purging, restricting). The prevalence of BED is 1–3% in older children and adolescents [159]. Less is known about children less than 10 years old. Twice as many girls are diagnosed with BED compared to boys [159]. However, BED has the highest prevalence of any eating disorder in males. The average age of onset usually in late adolescence. Risk factors for BED include weight-based teasing/bullying, sexual and gender minority youth, body dissatisfaction, and adverse childhood experiences [160,161]. Obesity with BED is associated with earlier onset overweight, more severe obesity, early onset of dieting, anxiety, depression, and ADHD [162].

Patients at high risk or exhibiting symptoms of excessive overeating are screened for BED. Screening instruments include: Adolescent Binge Eating Questionnaire [163] and the Sick, Control, One, Fat, Food (SCOFF) Questionnaire [164], which have high sensitivity for detecting BED in adolescents. Criteria for positive BED diagnosis includes episodes occurring at least one time per week for at least three months [165]. Binge eating episodes are associated with more than three of the following in a positive diagnosis [159,166]:

• Fast paced eating
• Eating until uncomfortable
• Eating large amount of food in the absence of hunger
• Eating alone because embarrassed by portions
• Feelings of guilt and shame after BED episode

The goals of BED treatment include [159]:

• Reducing binge eating episodes
• Decreasing the risk of medical complications
• Managing or reducing the risk of additional psychiatric complications

Treatment options include psychotherapy and pharmacotherapy, either additive or combined. Psychotherapy options include cognitive behavioral therapy, interpersonal psychotherapy, dialectical behavior therapy, and dialectic therapy [167]. Pharmacotherapy (topiramate, SSRIs, and lisdexamfetamine) may also help with weight loss, and can be useful in treating BED as well as depression and ADHD if they co-exist in the patient with BED [151,168].

### 4.2 Bulimia nervosa

Bulimia nervosa (BN) is defined as an obsessive desire to lose weight and is typically characterized by overeating followed by self-induced vomiting [169]. BN can occur at any weight across the spectrum of body habitus and BMI percentile. The prevalence of BN is 0.1–2% in children/adolescents with the average age of onset in late adolescence [170]. BN is more common in females than males. Risk Factors for BN include weight-based teasing/bullying, sexual and gender minority youth, emotional trauma and physical and/or sexual abuse. Teens with obesity are twice as likely to engage in unhealthy weight control behaviors (i.e., diet pills, laxatives, self-induced vomiting). BN has a strong association with mood (50%) and anxiety (66%) disorders; more than 50% of adolescents with BN report at least one episode of suicidal ideation [166,168,171].

Patients are screened if they are high risk or exhibit BN behaviors. The SCOFF screen [164] is commonly used. Clinical considerations when treating BN include identifying and managing any medical complications due to purging behaviors (ex. electrolyte imbalances or dysrhythmias), poor dentition and dental erosion, dyspepsia, gastrointestinal reflux disease, esophageal tears, colonic inertia, and any possible complications of these comorbidities (i.e., diabetes, kidney disease) [166,171].

Recurrent binge eating episodes are characterized by Refs. [172,173]:

• Eating a large amount of food in a short period of time (e.g., <2 hours)
• Feeling out of control or unable to stop eating
• Recurrent inappropriate compensatory behaviors to prevent weight gain
• Self-induced vomiting
• Misuse of diuretics, laxatives, or enemas
• Restricting or fasting
• Excessive exercise

The diagnosis of BN is made if the patient exhibits BN episodes and compensatory behaviors at least one time per week for at least three months along with body dissatisfaction and overvaluation of self and weight. Treatment involves a multidisciplinary approach to reduce binge-purge episodes, manage medical complications, and reduce the risk of psychiatric complications. Treatment options include [173,174]:

• Psychotherapy
• Cognitive behavioral therapy (CBT)
• Family-based behavioral therapy (FBT)
• Guided/unguided self-help interventions
• Pharmacotherapy

### 4.3 Night eating syndrome

First described in 1955, night eating syndrome (NES) is defined as the delay in the circadian intake of food with 25% or more of intake in evening or night [22]. The diagnosis is characterized by the patient awakening at least two times per week plus two of the following qualifiers [[175], [176], [177], [178]]:

• Morning anorexia
• Strong urge to eat between dinner and bed and/or night
• Sleep onset/insomnia at least 4 nights/week
• Belief that one must eat to get to sleep
• Mood frequently depressed and/or mood worse in evening
• Awareness and recall of eating during night
• Night eating present for 3 months

There is an increased risk for NES with obesity, other eating disorders, anxiety, depression, emotional or substance abuse, and insomnia. NES a risk factor for obesity. It is important to distinguish which eating disorder (or combination) is present in order to provide appropriate treatment. For example, although 7–24% of NES patients also meet the criteria for BED, the diagnoses are distinct from each other. BED patients eat large amounts of food in one sitting, but patients with NES eat smaller amounts throughout the night. Patients with BED score higher for emotional eating, body dysmorphia, and abnormal eating episodes as compared to NES. NES is associated with nocturnal anxiety, but BED is not [151,177,179].

NES also needs to be distinguished from sleep related eating disorder (SRED). In SRED patients, the timing of nocturnal eating occurs during slow wave sleep and there is no disturbance prior to falling asleep. SRED patients have partial or complete amnesia for eating episodes and 80% have associated comorbid sleep disorders [24]. SRED patients frequently consume non-food items and can injure themselves during eating/cooking. In contrast, NES occurs between dinner onward and during wakeful periods. Frequently NES patients have insomnia and can recall eating with episodes occur during wakefulness. NES patients eat food and are not at risk of injury [[179], [180], [181], [182]].

The diagnosis of NES is made using the Night Eating Diagnostic Questionnaire, which provides clinically useful diagnostic categories, and the Night Eating Questionnaire, which gives a global score for NES severity [183]. NES is a diagnosis of exclusion after ruling out substance abuse, other medical disorders, medication related complications, and psychiatric disorders. A sleep study can offer valuable information to guide therapy. Treatment for NES can include [134,184,185]:

• Anti-Depressants: SSRIs
• Cognitive Behavioral Therapy
• Melatonin
• Relaxation Techniques
• Bright Light Therapy
• Metabolic Bariatric Surgery

### 4.4 Sleep related eating disorder

Sleep related eating disorder (SRED) is defined as episodes of dysfunctional and involuntary eating and drinking that occur after a sleep arousal during the main sleep period and is associated with diminished levels of consciousness and subsequent recall. The prevalence is higher in young adult females; there is little data in the pediatric population. The exact mechanism of SRED is unknown, occurs more often in people with history of sleepwalking and stress, and is possibly linked to dopaminergic dysfunction [[179], [180], [181], [182]].

Medications may be associated with SRED and include zolpidem, triazolam, amitriptyline, olanzapine, risperidone, and antidepressants. There can be a family history of SRED or sleepwalking. The differential diagnosis for SRED includes nocturnal eating disorder, Kleine-Levin Syndrome, dissociative disorder, bulimia nervosa with nocturnal eating, and binge eating disorder. SRED can be associated with somnambulism, periodic leg movement syndrome, obstructive sleep apnea, depression, anorexia nervosa, BN, and BED [135]. Episodes of SRED occur in the first half of the night, often nightly, and include [180]:

• Eating and drinking in an out-of-control manner
• Impaired consciousness while preparing and eating food
• Little or no memory of these actions the next morning
• Eating high-carbohydrate and high-fat foods or odd combinations of food
• Possibly eating inedible or toxic substances, such as frozen foods, coffee grounds, cleaning solutions or cigarette butts
• Possibly experiencing injuries from engaging in dangerous food preparation activities or eating toxic substances
• Not being easily awakened or redirected during the episode
• Episodes can lead to excessive weight gain

Treatment includes for SRED includes [186]:

• Discontinue medications that can be triggers.
• Management of any associated sleep disorders such as sleepwalking, restless legs syndrome or obstructive sleep apnea.
• Safety strategies to get back to bed.
• Counsel and support a sleep routine for wake and sleep times, duration of sleep.
• Keep sleep area and kitchen safe
• Consider storing foods eaten during an episode outside the kitchen or placing locks on cabinets and the fridge
• Avoid alcohol and tobacco

Table 15 shows the top takeaways regarding disordered eating comorbidities associated with obesity in children.

**Table 15:** **Top takeaways: Disordered Eating Comorbidities Associated with Obesity in Children.** Shown are the top takeaways from the OMA regarding disordered eating comorbidities associated with obesity in children.

1.	Binge eating disorder (BED) is defined as excessive overeating that feels out of control, becomes a regular occurrence, and is without recurrent inappropriate compensatory behaviors (i.e. purging, restricting).
2.	Risk factors for BED include: weight-based teasing/bullying, sexual and gender minority youth, body dissatisfaction and adverse childhood experiences.
3.	Bulimia nervosa (BN) is defined as an obsessive desire to lose weight and is typically characterized by overeating followed by self-induced vomiting.
4.	The diagnosis of BN is made if the patient exhibits BN episodes and compensatory behaviors at least 1 time per week for at least 3 months along with body dissatisfaction and overvaluation of self and weight.
5.	Night eating syndrome (NES) is defined as the delay in the circadian intake of food with 25% or more of intake in evening or night.
6.	There is an increased risk for NES with obesity, other eating disorders, anxiety, depression, emotional or substance abuse, and insomnia.
7.	Sleep related eating disorder (SRED) is defined as episodes of dysfunctional and involuntary eating and drinking that occur after a sleep arousal during the main sleep period and is associated with diminished levels of consciousness and subsequent recall.

## 5 Conclusions

This Clinical Practice Statement on metabolic, behavioral health, and disordered eating comorbidities associated with obesity in children provides clinicians with recommendations regarding their pediatric patients. The interventions presented may lead to improvements in the health and wellbeing of children and adolescents with obesity, especially those with metabolic, physiological, and psychological complications.

### 5.1 Transparency [187]

This manuscript was largely derived and edited from the 2020–2022 Obesity Medicine Association (OMA) Pediatric Obesity Algorithm. Beginning in 2016, the OMA created and maintained an online Pediatric “Obesity Algorithm” (i.e., educational slides and eBook) that underwent updates approximately every two years by OMA authors and was reviewed and approved annually by the OMA Board of Trustees. Authors of prior years’ versions are included in Supplement #1. This manuscript is the first published version of the applicable chapter/s of the 2020–2022 OMA Pediatric Obesity Algorithm.

### 5.2 Group composition

Over the years, the authors of the OMA Pediatric Obesity Algorithm have represented a diverse range of clinicians, allied health professionals, clinical researchers, and academicians. (Supplement #1) The authors reflect a multidisciplinary and balanced group of experts in obesity science, patient evaluation, and clinical treatment.

## Author contributions

RK, DW, VO, JP, RC, SK, MC created and updated content and references of 2020–2022 OMA Pediatric Obesity Algorithm slides pertinent to this CPS. NTB transcribed from these slides the first draft of this CPS from the 2020–2022 OMA Pediatric Obesity Algorithm. SEC then reviewed, edited, and approved the document for pre-peer review submission and post-peer review publication.

## Disclosures (Declaration of potential competing interest)

Potential dualities or conflicts of interest of the authors are listed in the Individual Disclosure section. Assistance of a medical writer paid by the Obesity Medicine Association is noted in the Acknowledgements section. Neither the prior OMA Pediatric Algorithms nor the publishing of this Clinical Practice Statement received outside funding. The authors of prior OMA Pediatric Obesity Algorithms never received payment for their writing, editing, and publishing work. Authors of this Clinical Practice Statement likewise received no payment for their writing, editing, and publishing work. While listed journal Editors received payment for their roles as Editors, they did not receive payment for their participation as authors.

## Individual Disclosures

SEC declares a relationship with Novo Nordisk as a member of an Advisory Board and a relationship with Rhythm Pharmaceuticals as a member of their Gold Panel. NTB reports no disclosures pertaining to this project. VH reports no disclosures pertaining to this project. RK declares a relationship with Rhythm Pharmaceuticals: an ongoing funded study, two clinical trials, and membership in the RGDO Speaker Bureau. JP is a member of GOLD faculty and Rhythm Pharmaceuticals: Speaker's Bureau. RC declares a relationship with Rhythm Pharmaceuticals: Speaker's Bureau. SK declares a relationship with Abbott Nutrition: Speakers Bureau, relationship still active. DW reports no disclosures pertaining to this project. MC reports no disclosures pertaining to this project.

## Evidence

The content of the OMA Pediatric Obesity Algorithm and this manuscript is supported by citations, which are listed in the References section.

## Ethics review

After approval by the authors, a draft manuscript was peer-reviewed and approved by the OMA Board of Trustees prior to publication. This submission did not involve human test subjects or volunteers.

## Conclusions and recommendations

This Clinical Practice Statement is intended to be an educational tool that incorporates the current medical science and the clinical experiences of obesity specialists. The intent is to better facilitate and improve the clinical care and management of patients with pre-obesity and obesity. This Clinical Practice Statement should not be interpreted as “rules” and/or directives regarding the medical care of an individual patient. The decision regarding the optimal care of the patient with overweight and obesity is best reliant upon a patient-centered approach, managed by the clinician tasked with directing an individual treatment plan that is in the best interest of the individual patient.

## Updating

It is anticipated that sections of this Clinical Practice Statement may require future updates. The timing of such an update will depend on decisions made by Obesity Pillars Editorial team, with input from the OMA members and OMA Board of Trustees.

## Disclaimer and limitations

Both the OMA Obesity Algorithms and this Clinical Practice Statement were developed to assist health care professionals in providing care for patients with pre-obesity and obesity based upon the best available evidence. In areas regarding inconclusive or insufficient scientific evidence, the authors used their professional judgment. This Clinical Practice Statement is intended to represent the state of obesity medicine at the time of publication. Thus, this Clinical Practice Statement is not a substitute for maintaining awareness of emerging new science. Finally, decisions by practitioners to apply the principles in this Clinical Practice Statement are best made by considering local resources, individual patient circumstances, patient agreement, and knowledge of federal, state, and local laws and guidance.

## Declaration of competing interest

The authors declare the following financial interests/personal relationships which may be considered as potential competing interests: Suzanne Cuda reports a relationship with Rhythm Pharmaceuticals that includes: speaking and lecture fees and travel reimbursement. Suzanne Cuda reports a relationship with Novo Nordisk Inc that includes: consulting or advisory. Roohi Kharofa reports a relationship with Rhythm Pharmaceuticals that includes: funding grants and speaking and lecture fees. Jennifer Paisley reports a relationship with Rhythm Pharmaceuticals that includes: speaking and lecture fees and travel reimbursement. Rushika Conroy reports a relationship with Rhythm Pharmaceuticals that includes: speaking and lecture fees and travel reimbursement. Sara Karjoo reports a relationship with Abbott Nutrition that includes: speaking and lecture fees and travel reimbursement.
