# Supplementary material for: Metabolic, behavioral health, and disordered eating comorbidities associated with obesity in pediatric patients: An Obesity Medical Association (OMA) Clinical Practice Statement 2022
Source: Obes Pillars. 2022 Aug 6;3:100031. doi: 10.1016/j.obpill.2022.100031 (PMC10662000; doi:10.1016/j.obpill.2022.100031)
Supplement: Multimedia component 1 [file mmc1.docx]

**CSupplement #1: Historic Citation and Acknowledgement of Authorship of The Obesity Medicine Association Obesity Algorithms**

2020-2022 ebook Citation: Cuda S, Censani M, Paisley J, Browne N, O’Hara V. Pediatric Obesity Algorithm eBook, presented by the Obesity Medicine Association. [www.obesitymedicine.org/childhood-obesity](http://www.obesitymedicine.org/childhood-obesity)

2020-2022 Pediatric Algorithm slides: Cuda S, Censani M, O’Hara V, Browne N, Paisley J. Pediatric Obesity Algorithm, presented by the Obesity Medicine Association. [www.obesitymedicine.org/childhood-obesity](http://www.obesitymedicine.org/childhood-obesity). 2020-2022. [www.obesitymedicine.org/childhood-obesity](http://www.obesitymedicine.org/childhood-obesity)

2018-2020 Pediatric Algorithm slides: Cuda S, Censani M, Joseph M, Browne N, O’Hara V. Pediatric Obesity Algorithm, presented by the Obesity Medicine Association. www.obesitymedicine.org/childhood-obesity. 2018-2020. www.obesitymedicine.org/childhood-obesity

2016-2017: Cuda SE, Censani M, Joseph M, Green R, Scinta W. Pediatric Obesity Algorithm. (2016-17). Available online at: [www.Pediatricobesityalgorithm.org](http://www.pediatricobesityalgorithm.org/)
